# Depressive Symptoms in Individuals after Stroke in a Home-Based Rehabilitation Setting

**DOI:** 10.1155/2018/1621830

**Published:** 2018-04-11

**Authors:** Julianne Vermeer, Amanda McIntyre, Shannon Janzen, Danielle Rice, Laura Allen, David Ure, Robert Teasell

**Affiliations:** ^1^Lawson Health Research Institute, Parkwood Institute, London, ON, Canada; ^2^St. Joseph's Health Care, Parkwood Institute, London, ON, Canada; ^3^Department of Physical Medicine and Rehabilitation, Western University, London, ON, Canada

## Abstract

Poststroke depression has been shown to affect rehabilitation progress. This study evaluated patients after stroke who actively participated in a home-based rehabilitation program to determine variables that correlated with depressive symptoms in this population. A retrospective review of patients who were provided rehabilitation by Community Stroke Rehabilitation Team clinicians between January 1, 2009, and September 30, 2015, was completed. Logistic regression analysis was conducted to determine which demographic and outcome variables (Functional Independence Measure [FIM™] and Reintegration to Normal Living Index [RNLI]) were independently associated with depressive symptoms, as defined by Patient Health Questionnaire (PHQ-9) scores. 889 patients (53.2% male, mean age = 69.8 years) were included. Based on PHQ-9 scores, 89.7% of patients presented with no or mild depressive symptoms (PHQ-9 < 10) and 10.3% were considered to have moderate to severe depressive symptoms (PHQ-9 ≥ 10). The regression demonstrated that referral from outpatient, community care access centre, or community settings (OR = 1.89, *p* = 0.04), low RNLI scores (OR = 0.92; *p* = 0.001), and younger age (OR = 0.96; *p* < 0.001) predicted patients having moderate to severe depressive symptoms. Given the impact of poststroke depression on rehabilitation, clinicians should consider the potential impact of referral source, community reintegration, and age when monitoring and treating depressive symptoms.

## 1. Introduction

Worldwide, 15 million individuals have a stroke annually, of which five million are left with permanent deficits [1]. The physical impairments resulting from a stroke are often the main focus of treatment; however, psychological comorbidities are equally important as they influence a patient's recovery trajectory. The evaluation of symptoms of major depressive disorder is challenging as there is overlap in the criterion signs and symptoms between depression and stroke [2]. Poststroke depression (PSD) has been defined as “a prominent and persistent period of depressed mood or markedly diminished interest or pleasure in all, or almost all, activities that predominates in the clinical picture and that is thought to be related to the direct physiological effects of another medical condition [2].”

Depression is the most common neuropsychiatric disorder occurring after a stroke [3] with overall prevalence rates estimated to be 29% [4]. A meta-analysis reported rates to be 31% within the first 5 years, with rates slightly lower between 1 and less than 5 years (25%) [5], at five years (23%) [5], and at seven years after stroke (19%) [6] demonstrating that rates tend to decline over time. Robinson and Jorge [7] found that the frequency of PSD differed based on the clinical setting in which the patients were being treated and inferred that this was reflective of stroke severity. While Robinson and Jorge [7] report that individuals from community-based settings had lower depression prevalence rates compared to acute or rehabilitation hospitals or outpatient populations, Ayerbe et al. [4] found that the pooled prevalence of depression did not differ significantly over time or by clinical setting. Much of the literature to date has focused on individuals with a diagnosis of depression. However, it is worthwhile to note that many patients are treated with antidepressants or referred for psychotherapy that do not have a diagnosis of depression but rather the presence of symptoms [8]. There is large variability in rates of depressive symptoms among individuals after stroke (5–54%) [9, 10]. The large variability in depression diagnosis and depressive symptom rates may be reflective of the diverse measures, as well as inconsistencies in the cut-off scores, being used to determine PSD.

Most concerning, PSD has been found to negatively impact an individual's level of disability [4, 11], quality of life [4, 11, 12], mortality rate [4, 11], satisfaction with life, and utilization of rehabilitation services [11]. To effectively apply preventative measures and ensure that individuals are diagnosed and treated as soon as possible, Canadian Best Practice Recommendations for Stroke Care state that patients should be screened for depression at all points of care along the continuum [13]. Furthermore, there is a need to identify the factors associated with PSD as this information may also influence the referrals made for the individual and their recovery trajectory.

In general, the literature is conflicting in terms of predictors of PSD. The most commonly examined predictors have been age and gender; however, whether they are associated with depression varies considerably among studies [11, 14]. Stroke severity, dysphagia, incontinence, anxiety, social isolation, and living alone [4], as well as stroke lesion side [11], have been shown to predict depression. It is not known how these predictors vary by the setting in which individuals are assessed. That is, there is a need to further explore predictors of poststroke depressive symptoms, keeping in mind the population and setting being examined. Furthermore, a solid understanding of modifiable predictors, which are examined in this study, may allow for the direct translation of knowledge into clinical practice and promote early intervention.

## 2. Aims

The objective of this study was to evaluate individuals after stroke who participated in a home-based rehabilitation program to identify variables that correlate with depressive symptoms.

## 3. Methods

This study was granted ethics approval by the Western University Research Ethics Board in London, Ontario.

### 3.1. CSRT Program

The Community Stroke Rehabilitation Teams (CSRTs) provide home-based, multidisciplinary care to patients after stroke in Southwestern Ontario. Each patient receives an individualized rehabilitation plan based on their needs; services include physiotherapy (PT), occupational therapy (OT), speech language pathology (SLP), social work (SW), Registered Nursing (RN), therapeutic recreation specialist therapy (TRS), and rehabilitation therapy (RT). To be enrolled in the program, patients must have a stroke diagnosis and exhibit an ongoing need for rehabilitation and show motivation and the physical and cognitive capability to actively participate. Patients may be self-referred or be referred by a clinician or physician from any care setting at any point in the care continuum (e.g., acute, inpatient, and community).

### 3.2. Dataset

A retrospective review of patients receiving care between January 1, 2009, and September 30, 2015, was conducted. Data from the program was obtained from the CSRT's central administrative database, which was recorded directly by the program staff and clinicians.

### 3.3. Inclusion Criteria

For patients to be included in this retrospective analysis they must have met the following four a priori inclusion criteria: (1) actively received rehabilitation in the CSRT program (i.e., received ≥ 4 therapy sessions), (2) completed a Patient Health Questionnaire-2 (PHQ-2) depression screen, and (3) completed a Patient Health Questionnaire-9 (PHQ-9) depression screen if the patient had scored ≥ 3 on the PHQ-2.

### 3.4. Data Extraction

Extracted data included demographic variables (e.g., age, gender, marital status, type of stroke, and time after stroke) and program variables (e.g., referral source and number of therapy visits). Referral source was categorized as acute care; inpatient rehabilitation; outpatient, community care access centre (CACC or home care), or Community; and unknown. Admission scores from the following outcome measures were collected on patients during the first therapy visit by the appropriate therapist: PHQ-2 and PHQ-9 (SW and/or OT), Functional Independence Measure (FIM; PT and/or OT), and Reintegration to Normal Living Index (RNLI; OT).

### 3.5. Study Measures

*Patient Health Questionnaire (PHQ-2/PHQ-9)*. The PHQ-9 depression screen includes nine questions centred on a patient's feelings during a two-week period directly prior to the day of testing [15]. Patients self-administer or verbally answer each question on a scale from* not at all* (score = 0) to* nearly every day* (score = 3). Scores yield a maximum total of 27 and can be categorized as minimal (scores = 0–4), mild (scores = 5–9), moderate (scores = 10–14), moderately severe (scores = 15–19), or severe (scores = 20–27) depressive symptoms [15]. The PHQ-2 screen is a shortened version of the PHQ-9; it uses only the first two questions to determine if the patient warrants further screening for depressive symptoms.

Based on an established stepwise screening approach [16, 17], if patients scored ≥ 3 on the PHQ-2, they were further screened using the PHQ-9. A score of ≥3 shows high sensitivity and specificity for further depression diagnosis in the stroke population [18]. Patients displaying no symptoms to mild depressive symptoms (PHQ-2 score < 3 or PHQ-9 score of 0–9) were compared to those presenting with moderate to severe depressive symptoms (PHQ-9 score of 10–27). A cut-off score of 10 on the PHQ-9 was based on the high sensitivity and specificity reported in other studies with a stroke population [18, 19].

*Functional Independence Measure (FIM)*. The FIM indicates an overall level of independence in activities of daily living (ADLs) using cognitive and motor functioning and measures disability based on burden of care [20]. Scoring is based on 18 items, rated using a 7-point Likert scale ranging from* complete assistance *(score = 1) to* complete independence* (score = 7). High validity [21] and reliability [22] of the FIM have been established within a stroke population.

*Reintegration to Normal Living Index (RNLI)*. The RNLI is a measure of a patient's involvement in normal social activities, such as recreation and community participation, interaction with family or other relationships following a traumatic illness [23]. The index has 11 items that patients rate based on the degree of agreement with their personal situation. Each item is rated on a 3-point Likert scale (i.e.,* does not*,* partially does*, or* does agree*) to yield a total maximum score of 22 [23].

### 3.6. Data Analysis

Extracted data was entered into a Statistical Package for Social Sciences (SPSS; IBM, V22) database. Demographic statistics were calculated using frequencies and means with standard deviations. Prior to completing analyses, missing continuous data were analyzed using Little's Missing Completely at Random (MCAR) test to further determine if multiple imputation would be appropriate to apply in this dataset [24]. Missing continuous independent variables were replaced in the data set using multiple imputation. Multiple imputation uses case and group observed values within statistical algorithms to replace the missing values in a number of datasets as opposed to one. Ten imputations were applied as this has been found to provide sufficient efficiency of estimates [25]. These data sets were then pooled and used for the analysis in the logistic regression model [26].

A binary logistic regression was performed to evaluate the association of independent variables to patients with moderate to severe depressive symptoms. To determine appropriate variables to use within the regression model, preliminary independent sample *t*-tests were conducted for continuous variables (i.e., age, time post stroke, FIM, and RNLI scores) and chi-square tests of independence were conducted for categorical variables (i.e., gender, referral source, marital status, and type of stroke) to compare patients displaying no symptoms to mild depressive symptoms (PHQ 2 score < 3 or PHQ-9 score of 0–9) against those with moderate to severe depressive symptoms (PHQ-9 score of 10–27).

Based on the preliminary analyses, resultant significant variables were then further analyzed in the regression. Collinearity diagnostics were conducted to ensure that the assumption of multicollinearity was not violated. Independent variables used in logistic regression were age, referral source, and FIM and RNLI scores. The fit of the model was determined through the Hosmer-Lemeshow goodness-of-fit statistic whereby a larger *p* value indicates a good model fit. The Hosmer-Lemeshow statistic represents the accuracy of the predicted number of cases compared to the true number of cases. Further, the percentage accuracy in classification was an indication of the cases correctly classified by the model. Analysis was conducted using SPSS. Findings have been presented with odds ratios, (OR), 95% confidence interval (CI), and *p* values where statistical significance was set at *p* < 0.05, two-tailed.

## 4. Results

**Figure 1:**
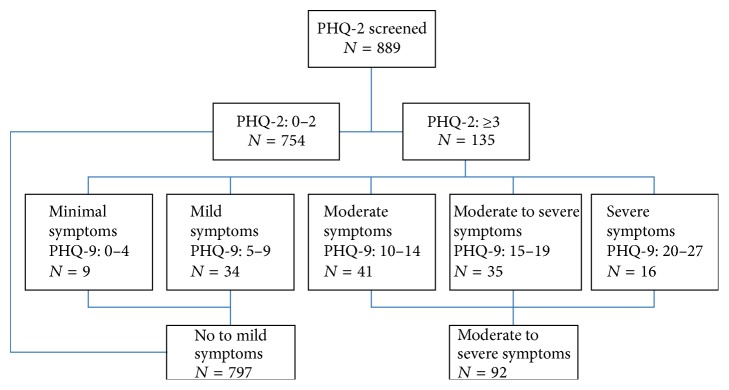
Depression screening flow diagram.

A total of 3,227 patients participated in rehabilitation in the CSRT program between January 1, 2009, and September 30, 2015. However, after applying the inclusion criteria, a large proportion of the sample was excluded. A total of 1,725 patients received fewer than four therapist visits and, therefore, were not considered to be receiving active rehabilitation and were excluded. An additional 613 patients did not receive complete PHQ screening and were excluded. Thus, just 889 patients could be included for analysis (Figure 1). There was no significant difference between those meeting or not meeting inclusion criteria on age (*p* = 0.062), gender (*p* = 0.865), type of stroke (*p* = 0.732), admission FIM scores (*p* = 0.389), or HADS scores (*p* = 0.986). The excluded group had fewer total therapy visits than the excluded group (*p* < 0.001) which is consistent with the application of the inclusion criteria.

Patients' ages ranged from 22 to 98 years (mean = 69.8 ± 13.0) where 53.2% were male and patients were on average 83.6 ± 200.5 days after stroke (median = 53.0). The majority of patients were referred from inpatient rehabilitation (49.6%), were married (61.1%), and had suffered an ischemic stroke (75.9%). Based on the depression screening, 89.7% (*n* = 797) of patients were considered to have had no to mild depressive symptoms and 10.3% (*n* = 92) were considered to have had moderate to severe depressive symptoms (Table 1).

Prior to completing the analyses, Little's MCAR test was performed to ensure a nonsignificant impact of missing variables, where chi-square results indicated that values were missing completely at random (*X*^2^ = 10.797, df = 9, *p* = 0.290). Preliminary analyses using *t*-tests and chi-square tests revealed significant differences between patients with no to mild depressive symptoms and those with moderate to severe depressive symptoms for age, referral source, FIM, and RNLI (*p* < 0.05, Table 2). There were no other significant between-group differences.

Sample size calculations were completed to ensure the sample size was adequate to perform the logistic regression with the specified independent variables (*N* = 10 × 5/0.10 = 500) [27]. After confirming the assumption of multicollinearity was not violated, age, patient referral sources, FIM scores, and RNLI scores were entered into the logistic regression model to determine association with moderate to severe depressive symptoms. Age and RNLI scores and the outpatient/CCAC/community referral source were each independent and significant predictors of moderate to severe depressive symptoms (*p* < 0.05, Table 3); FIM was not significant (*p* = 0.065). As a model, variables were able to distinguish between patients reporting moderate to severe depressive symptoms compared to those with no or mild symptoms, based on the Hosmer-Lemeshow test (*N* = 889, *χ*^2^ = 8.27, *p* = 0.41), indicating support for the model. The model correctly classified 89% of depressive cases.

The regression demonstrated that demographic factors and reintegration into normal living were independently associated with greater depressive symptoms. First, younger age was significantly associated with moderate to severe depressive symptoms (OR 0.96, C.I. 95%, 0.94, 0.97, *p* < 0.001). The referral source also impacted reports of depressive symptoms whereby those referred from outpatient/CCAC/community settings were more likely to have depressive symptoms (PHQ > 10) than those referred from an acute setting (OR 1.87, C.I. 95%, 1.02, 3.50, *p* = 0.04). There were no significant differences between referral groups for gender (*p* = 0.337) or time after stroke (*p* = 0.708). However, mean age (*p* < 0.001) and admission FIM scores (*p* < 0.001) were significantly different between referral groups. Those referred from outpatient settings were younger (66.0 years) compared to those from the community (67.9 years), acute care (68.4 years), inpatient rehabilitation (70.6 years), and CCAC (73.4 years). Those referred from CCAC had the lowest mean admission FIM scores (99.0) compared to those from inpatient rehabilitation (101.8), outpatient rehabilitation (106.7), the community (107.2), and acute care (110.8). Furthermore, patients' ability to reintegrate into normal life (RNLI) was significantly related to depressive symptoms; lower reintegration scores (OR = 0.90, C.I. 95%, 0.85, 0.96, *p* = 0.001) were associated with a greater odds of reporting depressive symptoms.

## 5. Discussion

Among a large sample of individuals receiving home-based stroke rehabilitation, this study found that the majority of people had no or mild depressive symptoms (~90%), as determined by the PHQ-2/PHQ-9. Individuals who were referred from outpatient/CCAC/community settings (*p* = 0.04), were younger (*p* < 0.001), or had lower RNLI scores on admission (*p* = 0.001) were more likely to have moderate to severe depressive symptoms (~10%). There was no difference between groups in gender, marital status, or type of stroke. Most notably, the logistic regression model was able to correctly classify 89% of depressive symptom cases.

PSD is a significant problem for many individuals. Prevalence rates are highly variable and have been shown to be correlated with several patient sociodemographic and clinical characteristics including timing, setting, and method of assessment after stroke, stroke type, and lesion side. The time at which patients are assessed and the setting in which they are assessed influence the extent to which depression is reported. For example, Matsuzuki et al. [28] completed assessments of depression among 117 patients (<4 weeks after stroke) from an acute care setting. The authors reported a depression prevalence rate of 68.4% on admission which declined to 56.4% at discharge. These findings are in contrast to the current study which assessed patients from the community who were, on average, 3 months after stroke and found a significantly lower prevalence rate (~10%). A systematic review by Bhogal et al. [29] examined the association between PSD and time since stroke but reported conflicting findings among studies as to whether there was any association.

Depression rate variability may also relate to method of screening and/or assessment. There are a plethora of depressive symptom screens and assessments, many of which have been used to evaluate the presence of PSD including the Hamilton Depression Rating Scale, Beck Depression Inventory, Beck Hopelessness Scale, Hospital Depression and Anxiety Scale, and “clinical judgement” [29]. Some inconsistencies in cut-off used with the PHQ-9 also produces variability in resultant prevalence of depressive symptoms. The current study chose to use a cut-off value of 10 for PHQ-9 scores due to its high sensitivity (0.80, 95% CI, 0.62–0.98) and specificity (0.78, 95% CI, 0.72–0.85) when used in the stroke population [30] but other studies have used other cut-off values [15]. A study by De Man-Van Ginkel et al. [30] reported a 12.2% prevalence of depressive symptoms among individuals 6 to 8 weeks after stroke, using a similar cut-off score of 10; notably, this rate is in line with our study.

The current study reported that individuals who had moderate to severe depressive symptoms were younger than those with mild or no symptoms. These findings are in contrast to a recent systematic review which reported that, among sixteen studies, thirteen found no association between PSD and age whereas the remaining three reported a higher prevalence of PSD among older adults [11]. This same relationship was reported in an earlier integrative review on PSD [31]. Other suggestions for differences between older and younger individuals point to generational health expectations over the long term. Research has suggested that, in general, older adults are more likely to have experienced more cumulative negative events in their lifetime and, therefore, have a well-rounded perspective on health challenges. A stroke is a significant life event that carries the potential for physical, cognitive, and psychosocial disability; older adults may have a greater ability to accept and adjust as a result of greater life experiences [32]. It was found that outpatients in the current study were younger than individuals referred from other settings. This age factor may help to explain why this community group was found to have greater levels of depressive symptoms; perhaps they did not have the same amount of coping abilities or the resulting impairments of stroke had a more substantial impact on their lives. Regardless, individuals face different life challenges depending on their life stage; programs, support groups, and resources, in general, should be customized to the age of the individual needing assistance.

Despite the fact that some studies have found no association between PSD and functional recovery [28], overwhelming evidence suggests that physical functioning after stroke is affected by depression [11]. The current study found that FIM scores were significantly different between the two groups but FIM was not significant when entered into the regression model. This could mean that other variables in our regression model partly account for the relationship between functional recovery and the presence of depressive symptoms. In examining change in depression status and FIM scores over time, Hadidi et al. [33] reported that, among individuals recruited from acute rehabilitation, FIM scores improved from baseline up to 1 month but that these scores plateaued between 1 months and 3 months after stroke. Similarly, depression improved between baseline and 2 weeks after stroke, but plateaued thereafter. Individuals without depression had higher FIM scores, and therefore better functional status, than those who were depressed. The stabilization of depression and FIM scores may reflect a transitional stage whereby patients respond to the shock of their stroke and undergo intense rehabilitation, improving ADLs and overall mood [33]. However, upon discharge, there are often fewer follow-up rehabilitation services available. Patients must then readjust to their home and surrounding community and this may reignite emotional disturbances [34]. Depression rates have been reported to be as high as 63% among individuals at 3 months after stroke, a time when the majority of people have discharged back to the community and may only be accessing health care as an outpatient [31]. This may help to explain why, in the current study, outpatients and individuals being referred from the community had significantly higher reports of depressive symptoms compared to those from acute care settings.

Functional ability and one's ability to reintegrate into their community are tightly linked. Murtezani et al. [35] reported that reintegration to normal living, as measured by RNLI, was related to functional outcome among 44 chronic stroke patients receiving rehabilitation whereby those with poorer reintegration had poorer outcomes in daily activity and quality of life. These findings are consistent with the current study, where individuals with lower FIM scores had lower RNLI scores, and were more likely to have moderate to severe depressive symptoms. Patients with less “primary effects” of the stroke (i.e., deficits such as poor physical functioning, immobilization) can more easily reintegrate into the community and participate in recreational or vocational activities as they face fewer barriers than individuals who require adaptive services and/or equipment [36]. In addition to the primary deficits following a stroke, psychological and social factors act as major barriers to community reintegration after stroke [36]. Individuals with PSD have been shown to experience social isolation thereby inhibiting their ability to interact with others in the home or community [37]. Implementing programs and applying interventions which aim to address depressive symptoms such as apathy, loss of interest in activities, loss of energy, irritability, and hopelessness may assist patients in transitioning back into their regular, formal, or informal, community activities.

There are limitations to the current study that warrant mentioning. First, this study used retrospective data from a large administrative database; the large number of missing PHQ screens and its impact on study outcomes demonstrate the limitations of using such a database. Second, depression screen scores were collected on patients at a single point in time during the home-based rehabilitation program. Since cross-sectional analysis does not allow for one to draw longitudinal conclusions, this is a potential avenue for future research. Finally, the PHQ-2/PHQ-9 was used to identify patients with no to mild depressive symptoms versus moderate to severe depressive symptoms. This tool does not formally diagnose patients with depression. However, a significant study performed by Williams et al. [19] demonstrated that, among 316 individuals following stroke, the PHQ-9 was able to discriminate well between individuals with and without major depression (area under the curve = 0.96); further, as indicated previously, a PHQ-9 cut-off score of ≥10 has excellent sensitivity and specificity in the poststroke population.

## 6. Conclusion

This study demonstrated that individuals with younger age, lower RNLI scores, or being referred from outpatient/CCAC/community settings were at increased odds of reporting moderate to severe depressive symptoms; these findings are in alignment with other studies. This research demonstrates the need for clinicians to continuously screen patients for depressive symptoms so that they are appropriately managed, given the functional and social ramifications of PSD.

## Figures and Tables

**Table 1:** Demographic and program descriptive variables for CSRT patients.

Variables	All patients *N* = 889		None to mild depressive symptoms^1^ *N* = 797		Moderate to severe depressive symptoms^2^ *N* = 92	
*Marital status, N (%)*						
Single	97	(10.9%)	84	(10.5%)	13	(14.1%)
Married/common law	543	(61.1%)	484	(60.7%)	59	(64.1%)
Divorced/separated	47	(5.3%)	41	(5.1%)	6	(6.5%)
Widowed	138	(15.5%)	130	(16.3%)	8	(8.7%)
Other/unknown	64	(7.2%)	58	(7.3%)	6	(6.5%)
*Gender, N (%)*						
Male	473	(53.2%)	428	(53.7%)	45	(48.9%)
Female	415	(46.7%)	368	(46.2%)	47	(51.1%)
Unknown	1	(0.1%)	1	(0.1%)	0	(0.0%)
*Mean age, years ± SD*	69.8 ± 13.0		70.5 ± 12.7		63.4 ± 13.7	
*Referral source, N (%)*						
Acute	293	(33.0%)	266	(33.4%)	27	(29.3%)
Inpatient rehab	441	(49.6%)	402	(50.4%)	39	(42.4%)
Outpatient Rehab/community/CCAC	154	(17.3%)	128	(16.1%)	26	(28.3%)
Unknown	1	(0.1%)	1	(0.1%)	0	(0.0%)
*Type of stroke, N (%)*						
Ischemic	675	(75.9%)	600	(75.3%)	75	(81.5%)
Hemorrhagic	113	(12.7%)	104	(13.0%)	9	(9.8%)
Unknown	101	(11.4%)	93	(11.7%)	8	(8.7%)
*Mean time following stroke,* *Days ± SD*	83.6 ± 200.5		81.1 ± 206.2		104.8 ± 141.3	

*Note*. ^1^PHQ-9 scores 0–9; ^2^PHQ-9 score ≥ 10; CCAC = community care access centre.

**Table 2:** Preliminary analyses of potential predictor variables for inclusion in regression model.

Variables	Depressive symptom groups		Independent samples *t*-tests	
	PHQ-9 0–9 (*n* = 797)	PHQ-9 ≥ 10 (*n* = 92)	*t*	*p*
Mean age, years ± SD	70.5 ± 12.7	63.4 ± 13.7	5.032	0.001
Time post stroke, days ± SD	81.1 ± 206.2	104.8 ± 141.3	−1.063	0.288

FIM, score ± SD	105.8 ± 17.0	99.4 ± 20.1	3.218	0.001
RNLI, score ± SD	15.9 ± 4.2	12.9 ± 4.8	5.233	0.001

			*Chi-square test of independence*	
			*X* ^2^	*p*

*Gender, N males*	428	45	0.781	0.377
*Referral source, N*			8.746	0.013
Acute care	266	27		
Inpatient rehab	402	39		
Outpatient Rehab/CCAC/community	127	26		
Unknown	0	0		
*Marital status, N*			4.475	0.215
Single	84	13		
Married	484	59		
Divorced/separated	41	6		
Widowed	130	8		
*Type of stroke, N*			1.757	0.415
Ischemic	600	75		
Hemorrhagic	104	9		
Unknown	93	8		

*Note*. CCAC = community care access centre; FIM = Functional Independence Measure; RNLI = Reintegration to Normal Living Index.

**Table 3:** Logistic regression model using pooled data from multiple imputation.

Variable	*B*	SE	*p*	Odds ratio	95% CI for odds ratio	
					Lower	Upper
*Age*	−0.044	0.009	0.001	0.957	0.941	0.973
*Referral source (reference = acute)*						
Inpatient rehab	−0.252	0.283	0.372	0.777	0.446	1.352
Outpatient Rehab/CCAC/community	0.646	0.314	0.039	1.909	1.032	3.531
*Admission FIM*	−0.013	0.007	0.065	0.987	0.974	1.000
*Admission RNLI*	−0.106	0.032	0.001	0.899	0.845	0.957

*Note*. CCAC = community care access centre; FIM = Functional Independence Measure; RNLI = Reintegration to Normal Living Index.
